# Intranasal dexmedetomidine improves postoperative sleep quality in older patients with chronic insomnia: a randomized double-blind controlled trial

**DOI:** 10.3389/fphar.2023.1223746

**Published:** 2023-11-16

**Authors:** Jinghan Wu, Xingyang Liu, Chunyan Ye, Jiajia Hu, Daqing Ma, E. Wang

**Affiliations:** ^1^ Department of Anesthesiology, Xiangya Hospital, Central South University, Changsha, China; ^2^ National Clinical Research Center for Geriatric Disorders (Xiangya Hospital), Central South University, Changsha, China; ^3^ Division of Anaesthetics, Pain Medicine and Intensive Care, Department of Surgery and Cancer, Faculty of Medicine, Imperial College London and Chelsea and Westminster Hospital, London, United Kingdom

**Keywords:** insomnia, dexmedetomidine, sleep quality, total joint arthroplasty, odler patients

## Abstract

**Objective:** This study was determined to investigate the impact of intranasal dexmedetomidine (DEX) on postoperative sleep quality in older patients (age over 65) with chronic insomnia during their hospitalization after surgery.

**Design:** A randomized double-blind controlled trial was conducted to compare the effects of intranasal dexmedetomidine spray with a placebo group.

**Setting and Participants:** The study was carried out at Xiangya Hospital, Central South University. 110 participants with chronic insomnia were analyzed.

**Methods:** This trial enrolled older patients who underwent total hip/knee arthroplasty and randomized them to receive intranasal dexmedetomidine (2.0 μg/kg) or saline daily at around 9 p.m. after surgery until discharge. The primary outcomes were subjective sleep quality assessed with the Leeds Sleep Evaluation Questionnaire (LSEQ). The secondary outcomes included the objective sleep quality measured with the Acti-graph, the Pittsburgh Sleep Quality Index (PSQI), the Insomnia Severity Index (ISI). The other outcomes included the incidence of delirium, levels of inflammatory factors, visual analog scale (VAS) pain scores, postoperative opioid consumption, and treatment-related adverse events.

**Results:** 174 patients were screened for eligibility, and 110 were recruited and analyzed. The DEX group had significantly higher scores on both the LSEQ-Getting to sleep and LSEQ-Quality of Sleep at each time point compared to the placebo (*p* < 0.0001), The least squares (LS) mean difference in LSEQ-GTS score at T0 between placebo group and DEX group was 2 (95% CI, −1–6), *p* = 0.4071 and at T5 was −14 (95% CI, −17 to −10), *p* < 0.0001; The LS mean difference in the LSEQ-QOS score at T0 was −1 (95% CI, −4 to 1), *p* = 0.4821 and at T5 was −16 (95% CI, −21 to −10), *p* < 0.0001. The DEX group exhibited significant improvement in Total Sleep Time (TST), Sleep Onset Latency (SOL), and Sleep Efficiency (SE), at each time point after treatment compared to the placebo group (*p* < 0.0001). The PSQI and ISI scores in the DEX group were reduced after treatment (*p* < 0.001). No significant adverse events were reported with the use of dexmedetomidine.

**Conclusion and Implications:** This study demonstrates that intranasal administration of dexmedetomidine improves postoperative sleep quality in older patients with chronic insomnia who undergo surgery, without increasing the incidence of adverse effects.

**Clinical Trial Registration:** http://www.chictr.org.cn/, identifier ChiCTR2200057133

## 1 Introduction

Sleep disturbances, including insomnia, are common among older adults, with up to 36% prevalence reported in older Chinese adults (CAO et al., 2017). Osteoarthritis (OA) is a degenerative disease that affects many seniors globally, with approximately 31% of OA patients experiencing significant difficulty falling asleep and 81% experiencing poor sleep maintenance (PARMELEE et al., 2015). Sleep disturbances often worsen during hospitalization due to factors such as pain, noise, nursing care activities, and surgical stress responses, leading to severe sleep deprivation, increased arousal time, and poor sleep quality (BANO et al., 2014; WESSELIUS et al., 2018), and it needs to take months to recover (HERRERO-SANCHEZ et al., 2014; CHEN et al., 2016). Postoperative sleep disturbance is a well-recognized contributing factor to acute cardiovascular events, immune suppression, and neurological complications such as delirium (TODD et al., 2017; IRWIN, 2019; HAACK et al., 2020; HUANG et al., 2020; WANG et al., 2021). Preoperative sleep disorders also increase chronic opioid use and medical utilization after surgery (RHON et al., 2019). Besides, it can also lead to pain and functional limitations in the months following surgery (CREMEANS-SMITH et al., 2006). Therefore, it is essential to prevent and/or treat sleep disorders in surgical patients, especially in older adults, for whom some medications are ineffective or may cause tolerance, dependency, and side effects with prolonged use (RIEMANN et al., 2015).

Dexmedetomidine (DEX) is a highly selective α2 adrenoceptor agonist with sedative and analgesic properties (REARDON et al., 2013). Dexmedetomidine promotes normal non-rapid eye movement (NREM) sleep by activating an endogenous sleep-promoting pathway (WU XH. et al., 2016). Dexmedetomidine administration during surgery has been shown to improve postoperative sleep quality and reduce severe sleep disturbances (SONG et al., 2019). Infusion of dexmedetomidine during the first night after surgery in ICU patients has also been shown to increase total sleep time, increase N2 sleep, and improve sleep efficiency and quality (Nelson et al., 2003). Dexmedetomidine infusion via patient-controlled analgesia has been found to provide effective analgesia and improve postoperative sleep quality in patients undergoing hysterectomy (CHEN et al., 2017). However, the clinical studies mentioned above administered dexmedetomidine intravenously, limiting its use for sleep disorder treatment. Intranasal administration of drugs is a convenient and non-invasive method that allows drug absorption through the nasal cavity and entry into circulation (XIE et al., 2017). Dexmedetomidine administered through intranasal spray has been shown to achieve effective sedation and anxiolysis in patients (WANG et al., 2014). Therefore, our study aimed to evaluate the effectiveness of intranasal dexmedetomidine spray in improving postoperative sleep quality in older patients with chronic insomnia after total joint arthroplasty.

## 2 Methods

This study was a double-blind, randomized, placebo-controlled parallel trial approved by the Ethics Committee of Xiangya Hospital (approval number: 202101038) and registered at the Chinese Clinical Trial Registry (ChiCTR2200057133). The study was conducted at Xiangyaya Hospitlal, Central South University, between 28 February 2022 and 30 July 2022.

### 2.1 Patient recruitment

At the beginning of their hospital admission, patients were evaluated for eligibility. After obtaining written informed consent, eligible patients were enrolled in this study if they met the following inclusion criteria: 1) patients aged 65 years or older who underwent total hip/knee arthroplasty; 2) patients diagnosed with chronic insomnia according to the third edition of the International Classification of Sleep Disorders (SATEIA, 2014), which includes severe difficulties initiating sleep, maintaining sleep, and/or early morning awakenings with no ability to return to sleep, insomnia duration of at least three times a week for at least 3 months, and impaired daytime functioning. The experienced doctors collected medical history and made the final clinical diagnosis using the International Classification of Sleep Disorders, third Edition. Exclusion criteria included: 1) preoperative sick sinus syndrome, severe bradycardia (heart rate <50 beats/min) or second degree or higher atrioventricular block, permanent pacemaker implantation, severe heart failure or ejection fraction <30%; 2) severe liver and kidney dysfunction (Child-Pugh Grade B and C, CKD3-5); 3) central nervous system diseases; 4) postoperative admission to the ICU; 5) allergy to the study drugs; 6) severe dementia, low cognitive function, language disability, severe hearing or visual impairment, mental disorder, coma, or any other diseases that would prevent completion of the study.

### 2.2 Anesthesia and surgery

We conducted lumbar plexus-sciatic nerve block with ropivacaine hydrochloride for all patients before general anesthesia on the day of their surgery. Intravenous general anesthesia was then induced using sufentanil (0.3 μg/kg), etomidate (0.2 mg/kg), and cisatracurium (0.2 mg/kg). Respiration was controlled or assisted through tracheal intubation or laryngeal mask, and anesthesia was maintained using continuous infusions of remifentanil (0.1–0.20 μg·kg−1 min−1) and propofol (4–8 mg·kg−1 h−1). Infusion rates were adjusted to keep the bispectral index (BIS) (COVIDIEN, Medtronic Parkway, United States) valve between 40 and 60 and maintain hemodynamic stability. A standardized analgesic regimen was employed, consisting of patient-controlled analgesia with sufentanil 100 μg diluted into 100 mL (with a continuous dosage of sufentanil 0.02 μg·kg−1 h−1 and a bolus dose of 0.02 μg/kg, with a 15-min lockout interval), supplemented with parecoxib 40 mg twice daily and dezocine 10 mg as needed.

### 2.3 Randomization and intervention

After patients were discharged from the post-anesthesia care unit (PACU), they were randomly assigned to either the DEX group (intranasal dexmedetomidine 2 μg/kg) or the placebo group. Randomization was performed using SPSS 26.0 software (SPSS Inc., Chicago, IL, United States) in a 1:1 ratio. A pilot study was conducted with three doses (1.0, 1.5, and 2.0 μg/kg), and 2.0 μg/kg was found to be the optimal dose for inducing stable sleep without significant side effects, based on a previous study of intranasal DEX for perioperative sedation and analgesic treatment in adults (CHEN et al., 2017). The study drugs (either dexmedetomidine hydrochloride or the same volume of normal saline) were provided as a clear aqueous solution in the mucosal atomization device (0.1 mL/dose, Wuxi NEST Biotechnology Co, Co., China) and administered by a researcher. The patients in DEX group were given intranasal spray of dexmedetomidine at a dose of 2 μg/kg every night from 21:00 to 21:30 from the night after surgery to discharge. The researcher used the mucosal atomization device to extract total dose of dexmedetomidine (concentration: 100 μg/mL, Hengrui Medicine Co., Ltd., Lianyungang, Jiangsu, China), then sprayed alternately to the both nostrils, with 0.1 mL per nostril, and the same nostrils was given every 2 min. The placebo group was given the same volume and speed of normal saline by the same mucosal atomization device. Sleep quality was assessed using both subjective (questionnaires) and objective (acti-graph) assessments. All study personnel, healthcare team, and patients were blinded to the treatment group assignment. In case of an emergency, doctors who were on duty had the right to unmask the blinding to initiate proper treatment as necessary. Treatment-related adverse events were monitored until 30 min after intervention, including hypotension (systolic blood pressure <90 mmHg or diastolic blood pressure <50 mmHg for 5 min or more), hypertension (systolic blood pressure >160 mmHg or diastolic blood pressure >100 mmHg for 5 min or more), bradycardia (heart rate <50 bpm), tachycardia (heart rate >100 bpm), and respiratory depression (respiratory rate <8 bpm or SpO_2_ < 92%). Adverse events were treated as routine care and recorded. To ensure a conducive sleeping environment for patients, all patients were housed in the same sound-insulated general ward, and nocturnal interventions were minimized during the study nights.

### 2.4 Study outcomes

The primary outcome of this study was assessed through subjective measures of sleep quality, using the Leeds Sleep Evaluation Questionnaire (LSEQ), a standardized self-reporting instrument completed daily. Secondary outcomes included objective measures of sleep quality, using the Acti-graph, as well as scores on the Pittsburgh Sleep Quality Index (PSQI) and the Insomnia Severity Index (ISI), which evaluate sleep over a period of time, and need to complete two assessments during the study–once at admission and once at discharge. Additionally, the study evaluated the incidence of delirium, levels of inflammatory factors (IL-6, CRP, ESR), length of hospital stay after surgery, VAS pain scores, postoperative opioid consumption, treatment-related adverse events, and postoperative complications.

### 2.5 Assessments and measurements

Subjective sleep quality: Patients completed the LSEQ at 8:00 a.m. daily from postoperative day 1 until discharge to assess their sleep quality after each treatment. The LSEQ is a standardized self-reporting instrument that evaluates sleep quality daily. The questionnaire consists of four items: ease of getting to sleep (GTS), quality of sleep (QOS), ease of awakening from sleep (AFS), and alertness and behavior following wakefulness (BFW). Each item is scored from 0 to 100 using a visual analogue scale (100 mm), with higher scores indicating better sleep quality (PARROTT and HINDMARCH, 1978).

The PSQI and ISI were administered once at admission and once at discharge to assess changes in subjective sleep quality after continuous DEX treatment. In addition, the PSQI was used to evaluate subjective sleep quality 30 days after discharge via phone. The PSQI includes nineteen individual items that generate seven “component” scores: subjective sleep quality, sleep latency, sleep duration, habitual sleep efficiency, sleep disturbances, use of sleeping medication, and daytime dysfunction. The severity of sleep quality is based on the score of the seven areas. Each area is scored between 0 and 3, and the PSQI score is calculated as the sum of the seven areas’ scores. Scores range from 0 to 21, with higher scores indicating more sleep disturbance (BUYSSE et al., 1989). The ISI assesses the severity of sleep-onset and sleep maintenance difficulties, satisfaction with the current sleep pattern, interference with daily functioning, noticeability impairment, and degree of distress or concern caused. Each item is scored between 0 and 4, and the ISI score is calculated as the sum of the seven items’ scores. Scores range from 0 to 28, with higher scores indicating more severe insomnia (BASTIEN et al., 2001).

Objective sleep quality: The Acti-graph (wGT3X-BT, ActiGraph, LLC., Pensacola, FL, United States) is a sleep and activity monitor. Objective sleep was recorded using the Acti-graph from 8 p.m. to 8 a.m. on the first night of admission (T0), the first night of intervention (T1), and continuously monitored until the fifth night of intervention (T5).

Delirium: The 3D-CAM was administered twice daily to assess patients delirium from the first day after surgery until the day of discharge (NEUFELD and THOMAS, 2013).

Pain: The visual analog scoring scale (VAS; with 0 indicating no pain and 10 indicating the worst imaginable pain) was used to measure the pain intensity at 4 h, 24 h, 48 h, and 72 h after surgery.

Inflammatory biomarkers: Blood samples were collected at admission and discharge to measure levels of the circulating cytokine Interleukin-6 (IL-6), C-reactive protein (CRP), and erythrocyte sedimentation rate (ESR) as inflammatory biomarkers.

### 2.6 Statistical analysis

Sample size calculation: The sample size was determined based on the results of a preliminary study and a previous investigation. The latter study reported a mean difference of 10 points in LSEQ scores between groups with a standard deviation of 15 points (CORNU et al., 2010). We assumed that our intervention would induce a similar significant improvement in sleep quality with a 10-point difference in LSEQ scores between groups. With a standard deviation of 15 points, a type I error rate of 5%, and a statistical power of 90%, the required sample size for each group was calculated to be 49. To account for a potential 10% dropout rate, we recruited 54 patients for each group using PASS 15 software (NCSS LLC., Kaysville, UT, United States) with a two-sample *t*-test assuming equal variances.

Data analysis: All statistical analyses were performed using IBM SPSS version 26.0 (IBM Corp., Armonk, N.Y., United States) and GraphPad Prism 9.2.0. Wilcoxon, Chi-Square analysis and Fisher exact test were used to evaluate between-group differences with respect to baseline characteristics. Continuous variables were reported as mean ± SD if they followed a normal distribution, and as median (interquartile range) if not. Categorical data were presented as numbers (percentages).

Repeated measures ANCOVA, as implemented under mixed models, was applied with change from baseline as the dependent variable, and treatment (DEX), time, and the treatment multiplied by time interaction as independent variables, to examine the main and interaction effects of treatment (DEX) and time on both objective and subjective sleep indices. Hypotheses of specific interest-e.g., between-group differences at the individual timepoints and within-group changes over time-were tested by defining contrasts among the regression parameters; predicted mean difference (LS) are the adjusted values from this model. The treatment-by-visit interaction term was tested first. If significant, between-group differences at each timepoint were tested at *α* = 0∙05. If not significant, the treatment main effect was tested next. Otherwise, Bonferroni correction was applied at each timepoint, with *p*-values adjusted by multiplying the nominal *p*-value by the number of tests (truncated at 1∙0). A two-sided *p*-value of less than 0.05 was considered statistically significant.

## 3 Results

### 3.1 Study population

**FIGURE1:**
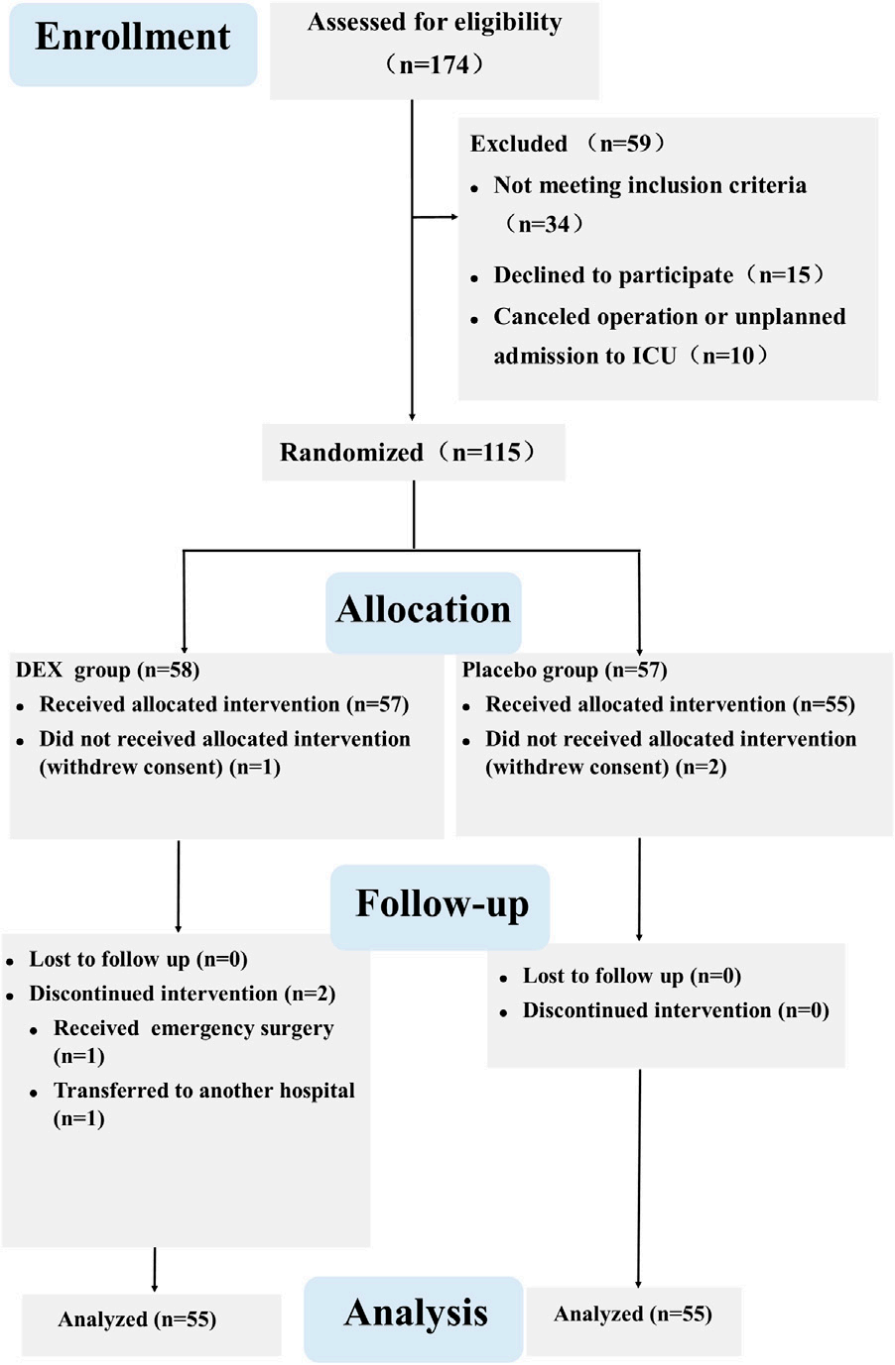
CONSORT flow diagram.

Out of 174 screened patients, 115 were ultimately randomized. We analyzed data from 110 participants (55 per group) for this report (Figure 1). A sample size of 115 patients who received the intervention was targeted to account for potential patient withdrawals. The ActiGraph data of 20 patients were not included in the analysis due to patient refusal or equipment problems (8 in the DEX group and 12 in the placebo group). There were no significant differences between the two groups in terms of patient characteristics and intraoperative parameters (Table 1).

**TABLE 1:** Baseline characteristics and intraoperative data.

	Placebo (n = 55)	DEX (n = 55)	*p-*value
Age median (IQR)	70 (67,73)	69 (67,74)	0.627
Sex(female,%)	36 (65.5%)	33 (60.0%)	0.554
BMI, kg/m^2^	23.57 ± 2.73	23.81 ± 2.92	0.525
Education, median (IQR),year	6 (6,9)	6 (5,9)	0.226
ASA classification			
II	18 (32.7%)	20 (36.4%)	0.688
III	37 (67.3%)	35 (63.6%)	
Preexisting conditions,n(%)			
Hypertension	26 (47.3%)	21 (38.9%)	0.377
Chronic cardiovascular disease	8 (14.5%)	6 (10.9%)	0.567
Diabetes	9 (16.4%)	8 (14.5%)	0.824
History of smoking,n(%)	8 (14.5%)	10 (18.2%)	0.606
History of drinking,n(%)	2 (3.6%)	6 (10.9%)	0.142
Surgical site			
TKA,n(%)	26 (47.3%)	28 (50.9%)	0.703
THA,n(%)	29 (52.7%)	27 (49.1%)	
Duration of surgery, min	112.84 ± 54.37	104.27 ± 36.97	0.337
Artificial airway			
Laryngeal mask insertion, n(%)	37(67.3%)	35(63.6%)	0.841
Tracheal intubation, n(%)	18(32.7%)	20(36.4%)	
duration of mechanical ventilation, min	140.73 ± 58.61	135.27 ± 41.40	0.057
Dosage of sufentanil, µg	26.73 ± 10.37	27.82 ± 11.85	0.325
Crystal liquid input, ml	687.27 ± 284.84	709.09 ± 259.11	0.675
Colloid input, median (IQR), ml	500 (500,1000)	500 (500,875)	0.419
Amount of blood loss, ml	220.55 ± 266.23	197.53 ± 173.13	0.592

Data are presented as the mean ± standard deviation (SD), the count (percentage), or median (IQR).

### 3.2 Sleep quality

**FIGURE 2:**
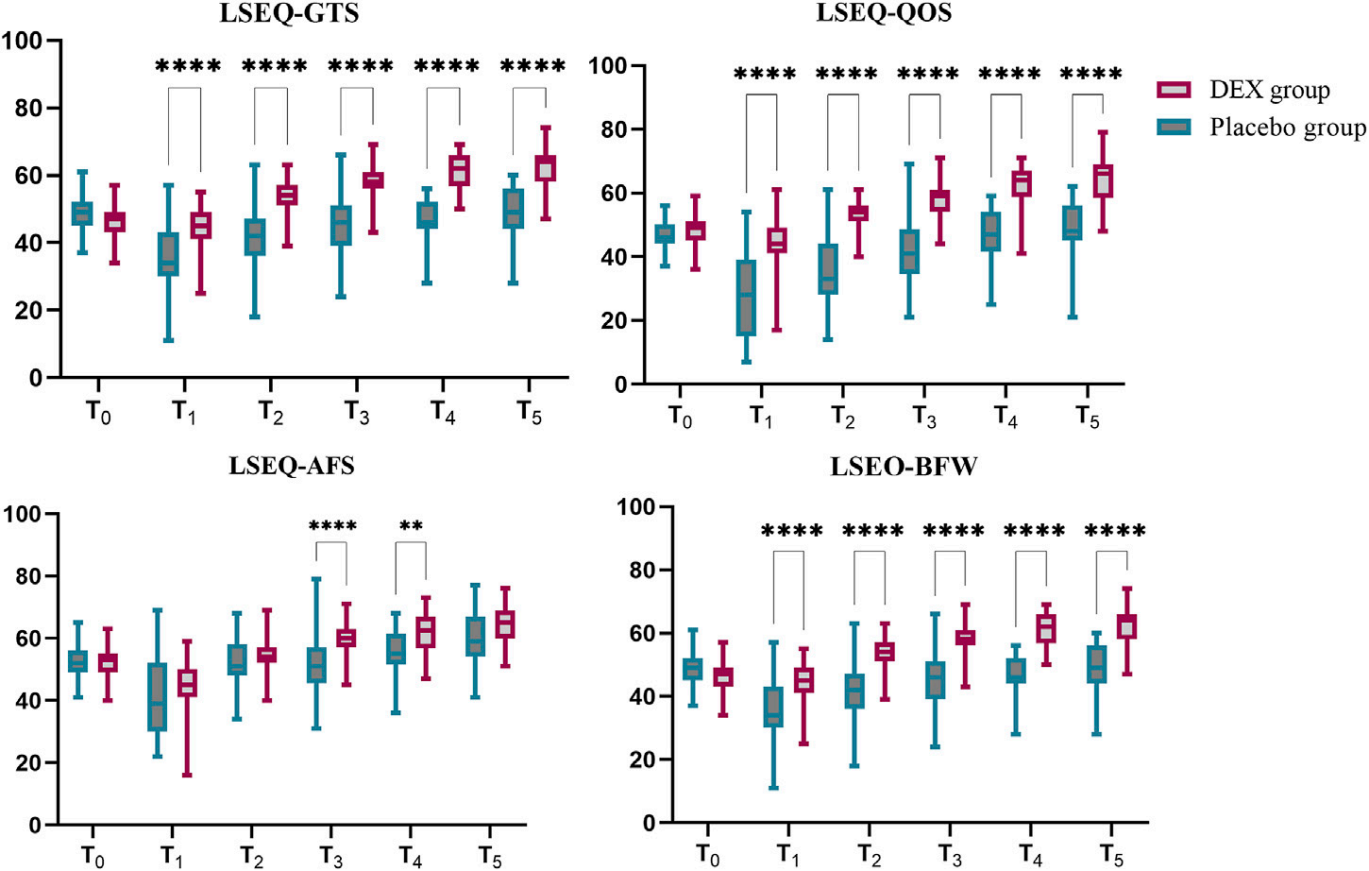
LSEQ scores of dexmedetomidine (DEX) and placebo groups. The LSEQ scores for “getting to sleep” (GTS) and “quality of sleep” (QOS) were significantly increased in the DEX group compared to baseline, while the scores consistently remained lower than baseline in the placebo group. In terms of “awakening from sleep” (AFS) and “behavior following wakefulness” (BFW), the placebo group scores were lower than those of the DEX group. The subjective improvements in sleep quality resulting from DEX treatment were reflected in easier sleep onset and better overall sleep quality.

Primary outcome: The primary end point was a change in LSEQ scores. The LSEQ scores for “getting to sleep” (GTS) and “quality of sleep” (QOS) were significantly increased in the DEX group compared to placebo. The least squares (LS) mean difference in LSEQ-GTS score at T0 between placebo group and DEX group was 2 (95% CI, −1–6), *p* = 0.4071 and at T5 was −14 (95% CI, −17 to −10), *p* < 0.0001; The LS mean difference in the LSEQ-QOS score at T0 between placebo group and DEX group was −1 (95% CI, −4 to 1), *p* = 0.4821 and at T5 was −16 (95% CI, −21 to −10), *p* < 0.0001.In terms of “awakening from sleep” (AFS) and “behavior following wakefulness” (BFW), the placebo group scores were also lower than those of the DEX group. The LS mean difference in the LSEQ-AFS score at T0 between placebo group and DEX group was 0 (95% CI, −2–3), *p* > 0.9999 and at T5 was −5 (95% CI, −9 to 1), *p* = 0.0878; The LS mean difference in the LSEQ-BFW score at T0 between placebo group and DEX group was 1 (95% CI, −2–3), *p* > 0.9999 and at T5 was −4 (95% CI, −8 to1), *p* = 0.0878.The subjective improvements in sleep quality resulting from DEX treatment were mainly reflected in easier sleep onset and better overall sleep quality (Figure 2, Supplementary Table S1).

**FIGURE 3:**
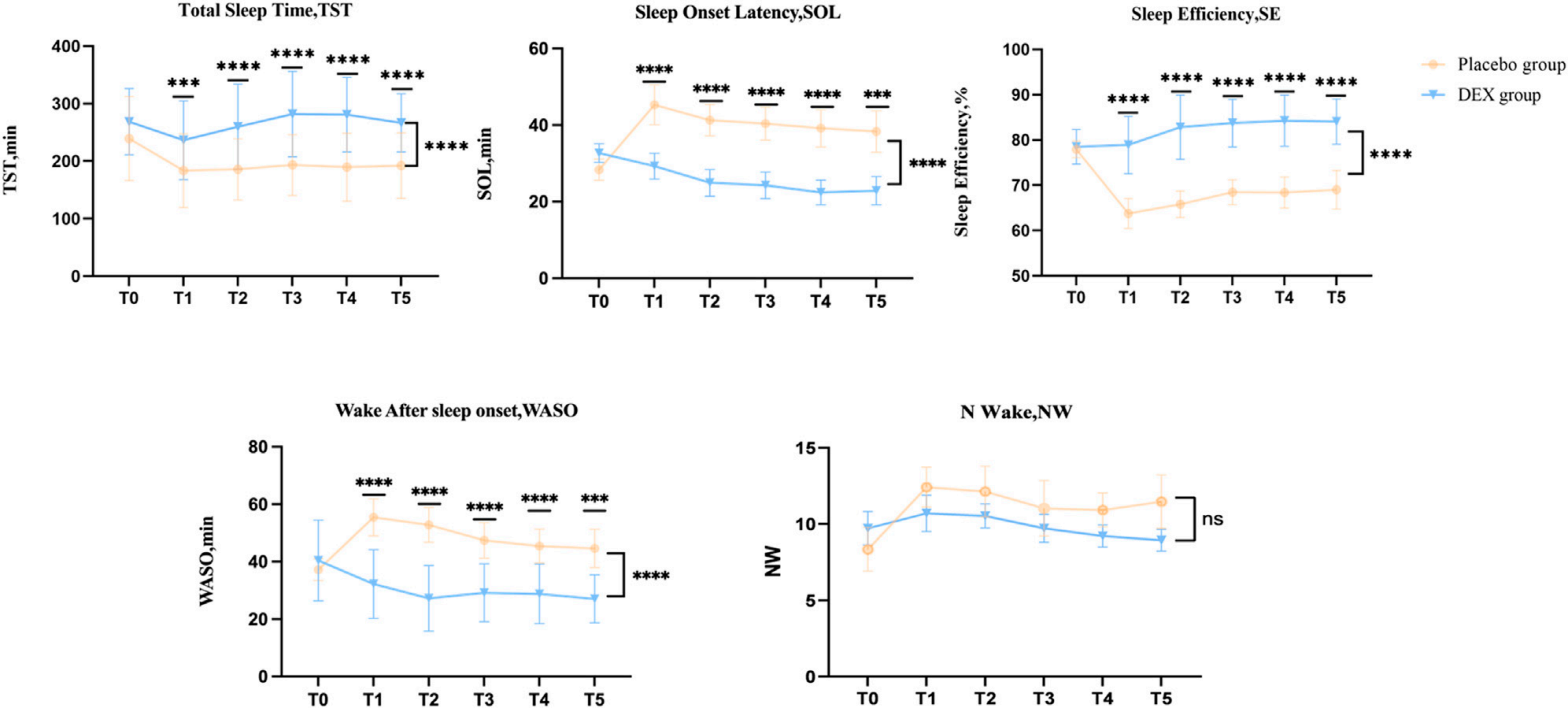
Acti-graph variables of dexmedetomidine (DEX) and placebo groups. The DEX group showed a significant increase in total sleep time (TST) compared to the placebo group, as well as a significant reduction in sleep onset latency (SOL) and wake after sleep onset (WASO). Dexmedetomidine treatment also significantly improved sleep efficiency (SE).

Secondary outcome:The baseline objective(acti-graph) sleep data were similar in both groups (Figure 3, Supplementary Table S2). After treatment, the DEX group showed a significant increase in total sleep time, sleep efficiency compared to the placebo group, as well as a significant reduction in Sleep Onset Latency (SOL) and Wake After Sleep Onset (WASO). The LS mean difference in Total Sleep Time (TST) at T0 between placebo group and DEX group was −29.23 (95% CI, –65.4 to 7.0), *p* = 0.4071 and at T5 was −87,8 (95% CI, −127.8 to −47.83), *p* < 0.0001; The LS mean difference in Sleep Onset Latency (SOL) at T0 between placebo group and DEX group was −4.3(95% CI, −9.3 to 0.7), *p* = 0.1277 and at T5 was 15.4 (95% CI, 6.6–24.2), *p* < 0.0001; The LS mean difference in Sleep Efficiency (SE) at T0 between placebo group and DEX group was −0.5 (95% CI, −3.5 to 2.4), *p* > 0.9999 and at T5 was −15.1 (95% CI, −21.3 to −8.8), *p* < 0.0001; The LS mean difference in Wake After Sleep Onset (WASO) at T0 between placebo group and DEX group was −3.2 (95% CI, −10.7 to 4.3), *p* > 0.9999 and at T5 was −17.6 (95% CI, 7.6–27.5), *p* < 0.0001; The LS mean difference in Number of awakenings (N-Wake) at T0 between placebo group and DEX group was −1.4 (95% CI, −3.8 to 1.0), *p* = 0.7641 and at T5 was 2.5 (95% CI, −0.1–5.1), *p* = 0.0583. (Figure 3, Supplementary Table S2).

The PSQI and ISI scores were decreased in the DEX group after treatment, indicating better sleep quality and relief of insomnia symptoms. In contrast, the placebo group had worse sleep quality and more severe insomnia symptoms during treatment. Furthermore, the PSQI score at 30-day after discharge was significantly lower in the DEX group compared to the placebo group (Table 2).

**TABLE 2:** Other outcomes between groups.

	Placebo (n = 55)	DEX (n = 55)	Difference (95%CI)	*p-*value
**Other sleep questionnaires scores**				
PSQI Admission score	10.60 ± 2.56	11.42 ± 2.97	−0.82(−1.87–0.23)	0.124
PSQI Discharge score	12.71 ± 2.32	9.04 ± 1.86	3.67(2.88–4.47)	<0.001
PSQI 30 days after discharge	12.49 ± 2.18	10.70 ± 2.11	1.79(0.98–2.60)	<0.001
ISI Admission score	10.96 ± 3.24	12.05 ± 3.43	−1.09(−2.35–0.17)	0.089
ISI Discharge score	12.65 ± 2.89	9.49 ± 2.73	3.16(2.10–4.23)	<0.001
**Inflammatory indicators levels**				
Pre-ESR, mm/h	28.00(15.00,50.00)	26.00(11.00,38.00)	1.00(−6.00–10.00)	0.724
Post-ESR, mm/h	49.00(34.00,72.00)	40.00(29.00,57.00)	8.20(0.00–18.00)	0.065
Pre-CRP, mg/L	5.24(3.14,8.30)	4.78(2.57,6.50)	0.88−−(−0.31–2.13)	0.137
Post-CRP, mg/L	62.3(31.70,93.40)	45.8(21.70,64.60)	16.03(4.10–30.50)	0.007
Pre-IL-6, Pg/mL^a^	6.64(2.41,9.80)	5.30(2.63,7.39)	0.83−(−0.93–2.80)	0.382
Post-IL-6, Pg/mL^a^	36.33(21.03,36.33)	21.80(11.60,43.03)	14.01(4.00–23.89)	0.006
**Postoperative pain VAS score**				
4 h	2.0(1.0,3.0)	2.0(1.0,3.0)	0.0(0.0–1.0)	0.480
24 h	4.0(4.0,5.0)	4.0(3.0,5.0)	0.0(0.0–1.0)	0.022
48 h	4.0(3.0,4.0)	3.0(3.0,4.0)	0.0(0.0–1.0)	0.075
72 h^b^	3.0(3.0,4.0)	3.0(3.0,3.0)	0.0(0.0–1.0)	0.034
Supplemental use of dezocine, n (%)	25(45.5%)	18(32.7%)		0.171
**Cognitive function**				
Delirium, n (%)	7(12.7%)	3(5.5%)		0.185
MMSE Admission score	26.0(23.0,27.0)	25.0(23.0,27.0)	0.0(−1.0–1.0)	0.911
MMSE Discharge score	25.0(23.0,27.0)	26.0(24.0,27.0)	−1.0(−2.0–0.0)	0.076
**Treatment-related adverse events**				
Respiratory depression, n (%)	0	0	-	
Hypertension, n (%)	2(3.6%)	4(7.3%)		0.675
Hypotension, n (%)	4(7.3%)	1(1.8%)		0.360
Tachycardia, n (%)	1(1.8%)	2(3.6%)		1.000
Bradycardia, n (%)	6(10.9%)	1(1.8%)		0.118
**Postoperative complications**				
Atrial flutter, n (%)	0	1(1.8%)		1.000
Dizziness, n (%)	11(20.0%)	14(25.5%)		0.495
PONV, n (%)	8(14.5%)	12(21.8%)		0.323
DVT, n (%)	0	2(3.6%)		0.475
Wound infection, n (%)	0	1(1.8%)		1.000
**PLOS**	5(4,6)	5(4,6)	0(0–1)	0.613

Data are presented as the mean ± standard deviation (SD), the count (percentage), or median (IQR).

^a^ There was some missing data for IL-6, N_P_ = 39; N_D_ = 45.

^b^ At 72 h after operation, some patients were discharged, N_P_ = 53; N_D_ = 55.

Abbreviations: PSQI: pittsburgh sleep quality index; ISI: insomnia severity index; VAS: visual analogue scale; MMSE: Mini-mental State Examination; PONV: postoperative nausea and vomiting; DVT: deep venous thrombosis; PLOS: postoperative length of stay.

### 3.3 Other outcomes

There was no significant difference in preoperative levels of CRP, IL-6, and ESR between the two groups (Table 2). However, postoperative levels of CRP and IL-6 were significantly lower in the DEX group compared to the placebo group, while ESR levels did not show a significant difference between the two groups (Table 2). There was no significant difference in pain VAS scores between the two groups, but the supplemental use of dezocine was slightly higher in the placebo group (Table 2). The incidence of delirium did not differ significantly between the two groups (Table 2). There were no significant differences in the incidence of treatment-related adverse events, including hypertension, hypotension, tachycardia, and bradycardia, as well as other postoperative complications, between the two groups (Table 2).

## 4 Discussion

Our data suggests that intranasal administration of dexmedetomidine can improve both the quality and efficiency of sleep while reducing postoperative levels of CRP and IL-6, without increasing the risk of adverse events. Previous studies have shown that intravenous DEX can improve sleep quality in surgical patients, but the effect of intranasal DEX on patient populations, especially older adults with chronic insomnia, has not been studied before.

Intranasal dexmedetomidine has been shown to provide satisfactory sedation with a favorable safety profile (JUN et al., 2017; KIM et al., 2017), even at a dose of 2 ug/kg, as used in our study and previous studies (WU et al., 2016b; UUSALO et al., 2019). After intranasal absorption, dexmedetomidine enters the central nervous system through the blood-brain barrier. It exerts its hypnotic action through activation of central pre- and postsynaptic α2-receptors in the locus coeruleus, thereby inducting a state of unconsciousness similar to natural sleep (AKEJU et al., 2018; HUANG et al., 2021). It is feasible to pharmacologically induce biomimetic N3 sleep with psychomotor sparing benefits. We believe that dexmedetomidine could be developed as a new class of sleep-enhancing medications with neurocognitive sparing benefits. It is worth noting that the benefits of using dexmedetomidine during the perioperative period for treating sleep disorders are not limited to this aspect. Dexmedetomidine’s analgesic and anti-inflammatory effects allow it to alleviate postoperative pain and reduce the risk of postoperative infection (UEKI et al., 2014; FLANDERS et al., 2019; MAO et al., 2020), both of which are risk factors for perioperative sleep disorders. This is consistent with our findings.

In addition, compared to intravenous administration, intranasal administration offers several advantages, including fewer adverse hemodynamic effects, non-invasive administration, and convenience (IIROLA et al., 2011; MILLER et al., 2018). Previous studies indicated that intranasal and intravenous dexmedetomidine had similar pharmacological effects (ZHANG et al., 2013). The drug absorption is slow after intranasal dexmedetomidine, and the peak concentration is significantly lower than that for its intravenous counterpart (IIROLA et al., 2011). Besides, absorption of intranasal medication is affected by many factors, includes mucociliary clearance rate, nasal secretion, and nasal mucosal blood flow, and administration volume, which might influence the bioavailability and pharmacological effect after intranasal treatment (WU et al., 2016c). This not only delays the drug onset time but also results in a less pronounced maximum effect of intranasal dexmedetomidine compared to intravenous. Therefore, the peak time of blood concentration of intranasal dexmedetomidine may be slow, and the peak concentration may be lower than that of intravenous dexmedetomidine. Our acti-graph data showed that dexmedetomidine decreased sleep latency and increased sleep efficiency after surgery, consistent with previous studies using intravenous infusion (ALEXOPOULOU et al., 2014; JIANG et al., 2018). Our study also found that dexmedetomidine treatment decreased postoperative CRP and IL-6 levels, indicating a reduction in perioperative inflammatory responses (DEL and GANGESTAD, 2018). This is consistent with previous studies demonstrating that dexmedetomidine administration inhibits surgery-induced inflammation and stress responses (WANG et al., 2019; CHEN et al., 2021). The anti-inflammatory properties of dexmedetomidine use after surgery highlight its unique advantage over other traditional sedative medications.

However, our study had some limitations, including the use of actigraphy instead of polysomnography to investigate sleep structure and the lack of conventional sleeping drugs as controls. The effectiveness of dexmedetomidine needs to be further studied in comparison to benzodiazepines.

Despite these limitations, intranasal dexmedetomidine is becoming an increasingly popular perioperative adjunct. Improving sleep quality is a shared objective for all healthcare providers, and multimodal sleep disorder treatment techniques will aid physicians in enhancing postoperative sleep management.

## 5 Conclusion

In summary, our study demonstrates that intranasal dexmedetomidine improves postoperative sleep quality in older patients with chronic insomnia who undergo total joint arthroplasty and are admitted to general wards, without significant adverse effects. This simple and convenient strategy has potential for widespread implementation in clinical practice. However, the relatively small sample size of our study warrants further investigation with a larger sample size to confirm its safety and efficacy.

## Data Availability

The original contributions presented in the study are included in the article/Supplementary Material, further inquiries can be directed to the corresponding author.
