# Supplementary material for: Intranasal dexmedetomidine improves postoperative sleep quality in older patients with chronic insomnia: a randomized double-blind controlled trial
Source: Front Pharmacol. 2023 Nov 16;14:1223746. doi: 10.3389/fphar.2023.1223746 (PMC10687473; doi:10.3389/fphar.2023.1223746)
Supplement: Supplementary file 1 [file Table1.docx]

Supplementary Table 1. LSEQ scores between groups

|  | | **GTS** | **QOS** | **AFS** | **BFW** |
| --- | --- | --- | --- | --- | --- |
| T0 | Placebo(n=55) | 49(45,52) | 48(44,51) | 52(49,56) | 48(44,52) |
|  | DEX(n=55) | 47(43,49) | 49(45,51) | 53(49,55) | 48(46,50) |
|  | Difference value, LS Mean (95% CI) | 2(-1 to 6) | -1(-4to1) | 0(-2to3) | 1(-2to3) |
|  | Adjust p*-*value | 0.4071 | 0.4821 | >0.9999 | >0.9999 |
| T1 | Placebo(n=55) | 34(30,43) | 29(17,40) | 39(30,52) | 37(33,43) |
|  | DEX(n=55) | 45(41,49) | 44(41,49) | 45(41,50) | 38(33.42) |
|  | Difference value, LS Mean (95% CI) | -8(-11to-4) | -16(-21to-10) | -3(-8to2) | 1(-3to5) |
|  | Adjust p*-*value | <0.0001 | <0.0001 | 0.6905 | >0.9999 |
| T2 | Placebo(n=55) | 42(36,47) | 36(29,46) | 51(48,58) | 48(43,52) |
|  | DEX(n=55) | 54(51,57) | 54(51,56) | 54(52,57) | 50(48,53) |
|  | Difference value, LS Mean (95% CI) | -13(-17to-9) | -17(-21to-13) | -2(-5to2) | -2(-5to1) |
|  | Adjust p*-*value | <0.0001 | <0.0001 | >0.9999 | 0.4116 |
| T3 | Placebo(n=53) | 46(39,51) | 41(36,50) | 51(46,57) | 55(52,59) |
|  | DEX(n=55) | 58(56,61) | 59(54,61) | 60(57,63) | 60(57,62) |
|  | Difference value, LS Mean (95% CI) | -12(-15to-9) | -16(-21to-12) | -8(-12to-3) | -4(-7to-1) |
|  | Adjust p*-*value | <0.0001 | <0.0001 | <0.0001 | 0.0009 |
| T4 | Placebo(n=45) | 46(44,52) | 47(42,54) | 55(52,62) | 59(55,64) |
|  | DEX(n=46) | 62(57,66) | 55(52,62) | 63(57,67) | 66(69,69) |
|  | Difference value, LS Mean (95% CI) | -14(-18to-11) | -14(-19to-11) | -6(-10to-2) | -5(-8to-2) |
|  | Adjust p*-*value | <0.0001 | <0.0001 | 0.0020 | 0.0010 |
| T5 | Placebo(n=31) | 49(44,56) | 48(45,56) | 59(54,67) | 64(58,68) |
|  | DEX(n=37) | 64(58,66) | 66(59,69) | 65(60,69) | 68(62,70) |
|  | Difference value, LS Mean (95% CI) | -14(-17to-10) | -16(-21to-10) | -5(-9to1) | -4(-8to1) |
|  | Adjust p*-*value | <0.0001 | <0.0001 | 0.0878 | 0.0984 |
| **Treatment** | *F* | 93.28 | 132.30 | 12.54 | 7.33 |
|  | *p-*Value | <0.0001 | <0.0001 | 0.0006 | 0.0079 |
| **Time** | *F* | 115.30 | 150.10 | 109.00 | 352.80 |
|  | *p-*Value | <0.0001 | <0.0001 | <0.0001 | <0.0001 |
| **Treatment x Time** | *F* | 42.70 | 28.65 | 6.21 | 6.44 |
|  | *p-*Value | <0.0001 | <0.0001 | <0.0001 | <0.0001 |

Data are presented as median (IQR). T0: the first night of admission; T1: the first night of intervention; T2: the second night of intervention, and so on.

GTS: Getting To Sleep; QOS: Quality Of Sleep; AFS: Awakening From Sleep; BFW: Behavior Following Wakefulness

Supplementary Table 2. Acti-graph variables between group

|  | | **TST, min** | **SOL, min** | **SE, %** | **WASO, min** | **N Wake** |
| --- | --- | --- | --- | --- | --- | --- |
| T0 | Placebo(n=43) | 239.3±11.2 | 28.0(20.0,33.0) | 78.0(75.3,82.0) | 39.0(28.0,48.2) | 8.0(6.0,10.0) |
|  | DEX(n=47) | 268.4±57.7 | 31.0(26.1,37.0) | 78.1(76.4,80.7) | 39.0(29.0,50.3) | 10.0(7.0,11.0) |
|  | Difference value, LS Mean (95% CI) | -29.23(- 65.4 to 7.0) | -4.3(-9.3to0.7) | -0.5(-3.5to2.4) | -3.2(-10.7to4.3) | -1.4(-3.8to1.0) |
|  | Adjust *p-*value | 0.1991 | 0.1277 | >0.9999 | >0.9999 | 0.7641 |
| T1 | Placebo(n=43) | 183.3±64.0 | 42.0(34.0,56.0) | 62.8(56.8,71.6) | 54.5(38.0,73.0) | 11.8(9.4,14.2) |
|  | DEX(n=47) | 236.4±68.4 | 27.0(20.0,35.0) | 79.7(75.7,83.2) | 28.8(23.0,39.0) | 10.0(8.0,12.0) |
|  | Difference value, LS Mean (95% CI) | -53.0(-89.2to-16.7) | 16.0(7.7to24.4) | -15.1(-20.2to-10.0) | 23.2(13.3to33.1) | 1.7(-0.6to4.1) |
|  | Adjust *p-*value | 0.0007 | <0.0001 | <0.0001 | <0.0001 | 0.3161 |
| T2 | Placebo(n=43) | 185.7±53.1 | 41.0(33.0,47.0) | 65.1(58.6,69.7) | 55.0(38.0,67.0) | 10.8(9.6,13.2) |
|  | DEX(n=47) | 259.8±74.2 | 23.2(16.0,29.9) | 84.1(81.8,86.8) | 25.0(18.0,32.1) | 10.0(9.0,12.0) |
|  | Difference value, LS Mean (95% CI) | -73.9(-110.2to-37.7) | 16.4(8.9to24.6) | -17.0(-21.8to-12.20) | 25.5(16.2to34.8) | 1.6(-0.9to4.1) |
|  | Adjust *p-*value | <0.0001 | <0.0001 | <0.0001 | <0.0001 | 0.5087 |
| T3 | Placebo(n=43) | 193.3±52.8 | 41.0(31.0,47.3) | 67.5(63.8,73.7) | 48.0(30.0,64.7) | 9.0(7.0,12.0) |
|  | DEX(n=47) | 281.8±74.3 | 22.0(17.0,26.74) | 84.8(82.2,87.6) | 27.0(22.0,34.0) | 9.0(8.0,11.0) |
|  | Difference value, LS Mean (95% CI) | -88.5(-124.8to-52.3) | 16.1(8.7to23.5) | -15.3(-19.5to-11.0) | 18.2(9.0to27.4) | 1.3(-1.4to4.1) |
|  | Adjust *p-*value | <0.0001 | <0.0001 | <0.0001 | <0.0001 | >0.9999 |
| T4 | Placebo(n=36) | 189.5±58.9 | 39.35(25.5,50.3) | 66.9(61.0,79.0) | 43.0(30.7,58.4) | 10.8(8.4,13.2） |
|  | DEX(n=42) | 280.1±64.9 | 20.0(15.0,26.3) | 85.9(80.9,88.0) | 27.0(21.7,35.0) | 9.0(8.0,10.0) |
|  | Difference value, LS Mean (95% CI) | -96.2(-133.3to-59.12) | 16.7(8.9to24.6) | -15.8(-21.0to-10.7) | 16.6(7.6to25.7) | 1.7(-.01to3.5) |
|  | Adjust *p-*value | <0.0001 | <0.0001 | <0.0001 | <0.0001 | 0.0679 |
| T5 | Placebo(n=21) | 192.4±56.9 | 38.0(29.0,46.5) | 67.1(63.1,75.2) | 43.6(34.2,54.8) | 10.6(8.6,14.8) |
|  | DEX(n=33) | 266.4±50.6 | 21.0(14.7,29.3) | 84.3(81.2,88.0) | 25.0(20.4,33.0） | 9.0(7.5,10.0) |
|  | Difference value, LS Mean (95% CI) | -87,8(-127.8to-47.83) | 15.4(6.6to24.2) | -15.1(-21.3to-8.8) | 17.6(7.6to27.5) | 2.5(-0.1to5.1) |
|  | Adjust *p-*value | <0.0001 | 0.0001 | <0.0001 | <0.0001 | 0.0583 |
| **Treatment** | *F* | 33.76 | 32.32 | 100.40 | 40.45 | 1.93 |
|  | *p*-Value | ＜0.0001 | <0.0001 | <0.0001 | <0.0001 | 0.1685 |
| **Time** | *F* | 19.58 | 12.19 | 23.60 | 6.76 | 11.52 |
|  | *p*-Value | <0.0001 | <0.0001 | <0.0001 | <0.0001 | <0.0001 |
| **Treatment x Time** | *F* | 13.49 | 27.28 | 40.83 | 24.96 | 4.66 |
|  | *p-*Value | <0.0001 | <0.0001 | <0.0001 | <0.0001 | 0.0004 |

Data are presented as the mean ± standard deviation (SD), count (percentage), or median (IQR).

TST: Total Sleep Time; SOL: Sleep Onset Latency; SE: Sleep Efficiency; WASO: Wake After Sleep Onset; N Wake: Number of Awakenings.
